# *Wolbachia* Biocontrol Strategies for Arboviral Diseases and the Potential Influence of Resident *Wolbachia* Strains in Mosquitoes

**DOI:** 10.1007/s40475-016-0066-2

**Published:** 2016-02-02

**Authors:** Claire L. Jeffries, Thomas Walker

**Affiliations:** Department of Disease Control, Faculty of Infectious Diseases, London School of Hygiene and Tropical Medicine, Keppel Street, London, WC1E 7HT UK

**Keywords:** Mosquitoes, Arboviruses, Mosquito biocontrol, *Wolbachia* bacteria

## Abstract

Arboviruses transmitted by mosquitoes are a major cause of human disease worldwide. The absence of vaccines and effective vector control strategies has resulted in the need for novel mosquito control strategies. The endosymbiotic bacterium *Wolbachia* has been proposed to form the basis for an effective mosquito biocontrol strategy. Resident strains of *Wolbachia* inhibit viral replication in *Drosophila* fruit flies and induce a reproductive phenotype known as cytoplasmic incompatibility that allows rapid invasion of insect populations. Transinfection of *Wolbachia* strains into the principle mosquito vector of dengue virus, *Stegomyia aegypti*, has resulted in dengue-refractory mosquito lines with minimal effects on mosquito fitness. *Wolbachia* strains have now been established in wild *St. aegypti* populations through open releases in dengue-endemic countries. In this review, we outline the current state of *Wolbachia*-based biocontrol strategies for dengue and discuss the potential impact of resident *Wolbachia* strains for additional target mosquito species that transmit arboviruses.

## Introduction

Arboviruses that cause human disease are predominantly transmitted by mosquitoes. Although there are more than 80 different arboviruses, most human cases result from infection with dengue virus (DENV) and other closely related Flaviviruses. The genus *Flavivirus* also includes West Nile virus (WNV), Yellow fever virus (YFV), Zika virus (ZIKV) and Japanese encephalitis virus (JEV). These medically important arboviruses are transmitted by several species of Culicine mosquitoes (Table 1). It is estimated that 40 % of the world’s population live in areas at risk for DENV infection in more than 100 countries. Global DENV infections range from 100–390 million per year, with 100 million symptomatic infections leading to 12,500 deaths per year [1•]. Dengue is an epidemic disease that occurs in tropical areas of Southeast Asia and South America and has a significant impact on developing countries [2]. Rare cases have been documented in the USA and southern Europe, and dengue is ‘re-emerging’ mostly due to the expansion of the geographical range of the principal mosquito vector, *Stegomyia* (*St*.) (*Aedes*) *aegypti*, through globalization and climate change [3]. There are currently no effective vaccines for DENV, so supportive treatment is the only method to reduce morbidity or prevent mortality for infected patients. Prevention of DENV transmission relies on mosquito vector control but this has not seen much success in recent years. Attempts to reduce the larval breeding of *St. aegypti*, predominantly an urban species, over a large geographical area have been difficult to achieve. There is also a delay between implementing larval control and achieving an impact on the adult mosquito population already transmitting disease. When dengue epidemics occur, the usual response is to use outdoor space spraying of insecticides. Although this targets the adult mosquito, insecticide resistance has been problematic in many countries. The options for developing new mosquito control strategies can be classified as either suppression (reduce or eliminate the wild mosquito population) or replacement with mosquitoes that are unable to transmit disease. A recent novel approach is the use of endosymbiotic *Wolbachia* bacteria to prevent DENV from replicating within the mosquito. In recent years, *Wolbachia*-based biocontrol has emerged as a very promising method that is environmentally friendly, safe to humans and potentially cost effective [4]. The ‘eliminate dengue’ project (www.eliminatedengue.com) has shown that *Wolbachia* can prevent DENV transmission in mosquitoes without significant fitness costs.

**Table 1:** Culicine mosquito vectors, arboviruses they transmit at significant levels in field populations and natural resident *Wolbachia* strains

Mosquito species	Arbovirus transmission	Resident *Wolbachia* strain
*Stegomyia aegypti*	DENV, YFV, CHIKV ZIKV	–
*Stegomyia albopicta*	DENV, CHIKV	*w*AlbA, *w*AlbB
*Stegomyia africana*	YFV	–
*Stegomyia simpsoni*	YFV	–
*Aedes bromeliae*	YFV	Unnamed
*Aedes fluviatilis*	YFV experimentally	*w*Flu
*Culex tritaeniorhynchus*	JEV, RVFV, WNV	–
*Culex quinquefasciatus*	WNV, RVFV, SLEV	*w*Pip
*Culex pipiens*	WNV, RVFV, SLEV	*w*Pip
*Culex tarsalis*	WNV, WEEV	–
*Culex univittatus*	WNV, SINDV	–
*Culex theileri*	RVFV	–
*Culex annulirostris*	MVEV, JEV, RRV	–
*Culex nigripalpus*	SLEV	–
*Culiseta morsitans*	EEEV	–
*Culiseta melanura*	WEEV, EEEV, WNV	–
*Coquillettidia perturbans*	WNV, EEEV	Unnamed
*Haemagogus spegazzinii*	YFV	–
*Ochlerotatus sollicitans*	WNV, EEEV	–
*Ochlerotatus taeniorhynchus*	WNV, EEEV, VEEV	–
*Ochlerotatus serratus*	VEEV	–
*Ochlerotatus vigilax*	RRV	–
*Ochlerotatus dorsalis*	WNV, CEV	–
*Ochlerotatus melanimon*	WNV, CEV	–
*Ochlerotatus triseriatus*	LCV	–
*Psorophora confinnis*	VEEV	
*Mansonia titillans*	VEEV	Unnamed

*DENV* dengue virus, *YFV* yellow fever virus, *CHIKV* chikungunya virus, *ZIKV* zika virus, *WNV* West Nile virus, *EEEV Eastern equine encephalitis virus*, *JEV* Japanese encephalitis virus, *SLEV* St Louis encephalitis virus, *RVFV* Rift Valley fever virus, *WEEV Western equine encephalitis virus*, *SINDV* Sindbis virus, *MVEV Murray Valley encephalitis virus*, *VEEV Venezuelan equine encephalitis virus*, *RRV Ross River virus*, *CEV California encephalitis virus*, *LCV* La Crosse virus

### Natural *Wolbachia* Infections in Mosquitoes

*Wolbachia* is a genus of obligate intracellular alphaproteobacteria within the family *Rickettsiaceae* [5]. *Wolbachia* was initially found in *Culex pipiens* mosquitoes in 1924 [6]. Since then, many different strains of *Wolbachia* have been discovered in a wide range of invertebrate hosts, including many filarial nematode and arthropod species. A recent study estimated that *Wolbachia* naturally infects more than 65 % of arthropod species [7]. Natural *Wolbachia* infections are present in some mosquito species that transmit human pathogens including *Culex* (*Cx*.) *quinquefasciatus* and *Stegomyia albopicta* (*Aedes albopictus*) (Table 1). Additional *Wolbachia* strains have been discovered in minor mosquito vectors of YFV including *Aedes* (*Ae.*) *bromeliae* [8] and *Ae. fluviatilis* which is infected with the *w*Flu strain [9]. *Wolbachia* infections are also present in wild mosquito populations of *Coquilletidia perturbans* [10] and *Mansonia titillans* [11]. However, natural infections are absent from most *Anopheles* spp. (that transmit malaria), *St. aegypti* and *Cx. tritaeniorhynchus* (the major vector of JEV). *Wolbachia* strains are currently divided into eight (A–H) supergroups according to sequence information [5]. *Wolbachia* strains have the ability to manipulate host reproduction in order to enhance their own reproduction and transmission through an insect population. *Wolbachia* strains can induce various phenotypic effects in insects including parthenogenesis, feminization, male killing and cytoplasmic incompatibility [5].

### Cytoplasmic Incompatibility

In mosquitoes, *Wolbachia* strains can induce cytoplasmic incompatibility (CI), which results in the generation of unviable offspring when an uninfected female mates with a *Wolbachia*-infected male [5]. In contrast, *Wolbachia*-infected females can produce viable progeny when they mate with both infected and uninfected males, resulting in a reproductive advantage over uninfected females. The CI phenotype allows the maternally transmitted *Wolbachia* to efficiently invade host populations without being infectious or moving horizontally between individuals [5].

CI can also be the result when mating occurs between mosquitoes with two different, incompatible *Wolbachia* strains. This CI can occur bidirectionally if crosses of both males and females with each different *Wolbachia* strain are incompatible, resulting in embryonic death in both crosses. However, some crosses between mosquitoes with differing strains can instead lead to unidirectional CI, where a cross of males with strain ‘x’ and females with strain ‘y’ results in CI, whereas the reciprocal cross (males with strain y and females with strain x) allows the production of viable offspring. In this case, mosquitoes with *Wolbachia* strain x would have a reproductive advantage over those with strain y and strain x and would be predicted to successfully spread through the population, “sweeping over” strain y [12].

### Transinfection of *Wolbachia* Into Mosquitoes

The various phenotypic effects of *Wolbachia* strains on natural insect hosts have led to a range of ideas on how this could be applied for mosquito-borne disease vector control. One potential strategy was investigated in the 1960s using repeated releases of *Wolbachia*-infected male *Culex* mosquitoes to suppress wild populations using CI [13]. A later discovery of the *w*MelPop strain of *Wolbachia* in *Drosophila melanogaster* fruit flies, which dramatically lowered the lifespan of its host [14], led to the potential use of ‘life-shortening’ strains to manipulate the population age structure of important mosquito vectors. Recently, *Wolbachia* strains from *Drosophila* fruit flies were found to protect their native hosts against infection by pathogenic RNA viruses [15, 16]. *Wolbachia* strains are associated with a significant reduction in the viral density of a range of viruses in flies, which delays insect mortality [17].

The use of *Wolbachia* for mosquito biocontrol first required the stable infection (transinfection) of target mosquito species. The first target species selected was *St. aegypti*, the principle vector of DENV. The *w*AlbB strain of *Wolbachia* was successfully established in *St. aegypti* using embryo cytoplasm transfer from closely related *St. albopicta* mosquitoes [18]. *St. aegypti* lines were later stably transinfected with *w*MelPop and *w*Mel strains from the native host *Drosophila melanogaster* [19, 20]. These transinfected *Wolbachia* strains significantly reduced the vector competence of *St. aegypti* for DENV in laboratory experiments [20–22]. High levels of *Wolbachia* bacteria infected the tissues that play a crucial role in DENV replication within mosquitoes. The presence of infectious DENV in saliva was completely inhibited by *Wolbachia* [20].

Successful *Wolbachia*-based biocontrol would also require invasion of wild mosquito populations. *Wolbachia*-infected females must vertically transmit the bacteria to their progeny at a high frequency. All three transinfected *Wolbachia* strains (*w*AlbB, *w*Mel and *w*MelPop) show maternal transmission rates close to 100 % and induce high levels of CI in *St. aegypti* [18–20]. The ability of transinfected *Wolbachia* to successfully invade wild *St. aegypti* mosquito populations will depend on a balance between negative selection imposed by fitness costs of the bacteria on the mosquito and positive selection associated with CI induction. The *w*MelPop strain results in significant fitness costs (including impacts on adult longevity and fecundity) so was considered inappropriate for initial test releases into wild mosquito populations.

### The Release of *Wolbachia*-Infected Mosquitoes

The invasive potential of *Wolbachia* strains was first tested in a semi-field facility that simulated the natural habitat of *St. aegypti* in north Queensland, Australia [20]. Successful trials led to mosquitoes infected with the *w*Mel strain being released into the wild through open releases in two locations near Cairns in north Queensland, Australia [23]. The *w*Mel strain successfully invaded the two natural populations, infecting nearly 100 % of the local population within a few months following releases. Prior to the release of *Wolbachia*-infected mosquitoes in Australia, extensive engagement with the communities in the release areas took place to determine the attitudes and levels of knowledge about dengue and mosquitoes. The success of these initial trials has led to further releases in DENV endemic countries such as Indonesia, Vietnam and Brazil (www.eliminatedengue.com). Continued success of a release program will require maintenance of an inhibitory effect on DENV replication in wild *Wolbachia*-infected *St. aegypti* populations. Vector competence experiments carried out with field *w*Mel-infected mosquitoes, collected 1 year following field release, indicated insignificant DENV replication and dissemination [24•]. Currently, work is being undertaken to determine the optimal *Wolbachia* strain for applied use that can balance the effects on pathogen transmission and fitness costs to the mosquito. Recent mathematical models of DENV transmission incorporating the dynamics of viral infection in humans and mosquitoes predict that *w*Mel would reduce the basic reproduction number, R_0_, of DENV transmission by approximately 70 % [25]. At the current time, it remains unclear what effect *Wolbachia* will have on DENV transmission and dengue epidemiology in the field. A cluster-randomized trial is premature because the choice of *Wolbachia* strain for release and deployment strategies is still being optimized [26•].

### Potential Use of *Wolbachia* for Additional Arboviral Diseases

Although *Wolbachia*-infected *St. aegypti* were originally generated for biocontrol of dengue, they are likely to have the added benefit of reducing transmission of additional arboviruses vectored by this mosquito species, including chikungunya virus (CHIKV) [21] and YFV [27] and potentially ZIKV. Like *St. aegypti*, mosquito species that are the principle vector of arboviruses and contain no natural *Wolbachia* infections are likely to represent the most feasible transinfection targets. JEV is predominantly transmitted by *Cx. tritaeniorhynchus* mosquitoes, and a *Wolbachia*-based biocontrol strategy has the potential to reduce transmission if stably infected lines can be generated [28]. JEV is part of the same genus as DENV (*Flavivirus*), so *Wolbachia* strains would likely provide similar inhibitory effects on transinfected mosquitoes. *Drosophila Wolbachia* strains grow to high densities in their native and transinfected hosts and provide strong inhibition of both insect viruses in *Drosophila* [16] and DENV in mosquitoes [20]. Successful transinfection of *Drosophila Wolbachia* strains in *Cx. tritaeniorhynchus* and the release of stably infected mosquitoes which enable the *Wolbachia* strain to invade wild populations, with strong viral interference characteristics, would be likely to significantly reduce JEV transmission [28].

### Resident *Wolbachia* Strains in Mosquitoes and Effects on Arbovirus Replication

Some major mosquito vectors of arboviral diseases harbor natural *Wolbachia* infections. Both DENV and CHIKV are also transmitted by *St. albopicta*, which contain two resident strains of *Wolbachia*; *w*AlbA and *w*AlbB [29]. The principle vectors of WNV in the USA, *Cx. pipiens* and *Cx. quinquefasciatus*, contain a natural *Wolbachia* strain, *w*Pip. Minor differences in vector competence may be due to the presence of these resident *Wolbachia* strains. For example, *Cx. quinquefasciatus*, infected with the *w*Pip strain of *Wolbachia*, is generally less susceptible to WNV than *Cx. tarsalis* [30], which is not infected with *Wolbachia*. However, resident *Wolbachia* infections in mosquitoes do not impact arboviral transmission to the same extent as transinfected *Drosophila Wolbachia* strains, as reviewed in [28]. One mechanism suggested for this difference is that the newly transinfected strains trigger an immune response in their new invertebrate hosts, which also has antiviral effects, whereas native *Wolbachia* strains have been present in the host long enough that the host no longer generates such an immune response. However, the complexity in the interaction between *Wolbachia*, the insect host and arboviruses remains unclear [31].

Although the mechanism of how *Wolbachia* inhibits arboviruses is not fully known, *Wolbachia* density is correlated with viral interference in both native *Drosophila* [32] and transinfected *St. aegypti* [20]. In general, resident *Wolbachia* strains in wild populations do not appear to grow to such high densities as transinfected strains and therefore have less impact on arboviral vector competence. For example, a high-density *w*Pip strain in a laboratory colonized line of *Cx. quinquefasciatus* was observed to show resistance to WNV, compared to a *w*Pip cleared line [33]. However, lower density *w*Pip infections found in field-collected *Cx. quinquefasciatus* and *Cx. pipiens* mosquitoes do not appear to be capable of inhibiting WNV infection and transmission [34]. Recent studies have shown that the removal of resident *Wolbachia* strains from *St. albopicta*, followed by transinfection of the *Drosophila w*Mel strain, results in strong inhibition of both DENV and CHIKV [35, 36]. Therefore, the transinfection of mosquito species with *Drosophila Wolbachia* strains would likely provide strong inhibitory effects on arbovirus transmission.

### Resident *Wolbachia* Strains and Mosquito Population Invasion

The potential impact of resident *Wolbachia* strains on the ability of introduced transinfected strains must be considered if species such as *Cx. quinquefasciatus* and *St. albopicta* are considered targets for *Wolbachia* biocontrol. The first point to consider is whether the resident strain actually inhibits the ability to form stable transinfected lines with *Drosophila Wolbachia* strains. *Wolbachia* bacteria are maternally inherited so are found at high densities in the reproductive tissues (ovaries for females). As *Wolbachia* transinfection involves the injection of preblastoderm embryos for the infection of the pole cells (that form the germline), the presence of high-density resident *Wolbachia* strains is likely to decrease the chances of success. However, an artificial triple *Wolbachia* infection in *St. albopicta* was successfully created with the *w*Ri strain of *Wolbachia* from *Drosophila simulans* and yielded a new pattern of cytoplasmic incompatibility [37]. This generation of a ‘superinfected’ line with a resident strain and a transinfected strain has not been possible so far in *Cx. quinquefasciatus* (despite significant efforts).

There may be species-specific differences between *Wolbachia*-mosquito host interactions that impact transinfection success. A stable, *Wolbachia* superinfected line with new transinfected strains (*w*Mel and *w*AlbB) was recently generated in *St. aegypti* (Joubert et al. unpublished data). The transinfection options for target mosquito species such as *Cx. quinquefasciatus* that contains a single resident strain (*w*Pip) include removal of the resident strain through antibiotic treatment or attempting to create a resident strain/transinfected strain superinfection. If a single infection containing a novel transinfected *Wolbachia* strain (eg. *w*Mel) was generated, the crossing patterns induced between infected mosquitoes could still result in the invasion of the transinfected strain. Matings involving two different *Wolbachia* strains in *Cx. quinquefasciatus* would result in bidirectional CI [38] and two strains cannot stably co-exist in a given mosquito population. The strain that reaches the highest local frequency would likely reach fixation given that females infected with this strain would be at a reproductive advantage (more males to mate with that are compatible). However, if significant fitness costs are associated with a transinfected *Wolbachia* strain this would prevent the invasion of the population and replacement of the resident *w*Pip strain. 

The potential role of *Wolbachia* strains in the speciation and genetic evolution of mosquito populations over time through unidirectional or bidirectional CI patterns between different native or introduced strains should also be considered, in addition, to the resultant phenotypic effects [39]. Other forms of reproductive interference can also affect mosquito vector population dynamics and could therefore potentially impact on the spread of *Wolbachia*. For example, satyrization is a form of reproductive interference whereby males of one species mate with females of another, producing no viable offspring but leading to the mated female no longer being receptive to further insemination for the remaining life of that mosquito [40, 41]. This phenomenon is believed to result from male accessory gland products and has been linked to population displacement in certain areas of *St. aegypti* by *St. albopicta* [42]. As *St. albopicta* has a resident *Wolbachia* strain and *St. aegypti* does not, if CI of *Wolbachia* in *St. albopicta* is not itself involved in this mechanism, satyrization could potentially assist in or lead to the amplification of the driving force of *Wolbachia* into certain populations in a population replacement strategy.

In addition to the presence of resident *Wolbachia* strains, the presence of other microorganisms within the mosquito population is another important factor to consider, which could affect the introduction of any transinfected *Wolbachia* strain into that population. For example, the bacteria *Asaia* is stably associated with many mosquito species [43] and has been found to compete with *Wolbachia* within a host, seemingly preventing or reducing *Wolbachia* establishment. It has also been suggested that the presence of *Asaia* in certain mosquito species could be a reason for the absence of native *Wolbachia* strains in such species [44].

## Conclusion

*Wolbachia*-based biocontrol strategies are likely to provide an environmentally benign and effective long-term control option for arboviral diseases such as dengue. Invasion and maintenance of transinfected *Wolbachia* strains in natural mosquito populations is likely to be cost effective given this should be self-sustaining through the CI phenotype and maternal transmission. Although significant advancements have been made in the implementation of *Wolbachia* for dengue biocontrol, the optimal *Wolbachia* strain (or combination of strains) to balance the inhibitory effects of DENV and fitness costs to the mosquito is still to be determined. A major area of research that needs addressing is how *Wolbachia* inhibits arboviruses in mosquitoes as further knowledge would allow more accurate predictions of the evolutionary consequences of strong selection pressure on DENV. This would likely provide better estimates of the effect *Wolbachia* would have on dengue transmission. *Wolbachia*’s broad protective inhibition of human arboviruses suggests the potential of this strategy to reduce the transmission of other arboviral diseases. The presence of resident *Wolbachia* strains in some mosquito vectors must be considered if transinfection (with subsequent favourable phenotypic effects) is to be successfully implemented. If promising initial laboratory research and field trials can translate into effective biocontrol programs, *Wolbachia* may provide a novel method to control additional mosquito-borne diseases.
